# Early and Midterm Outcomes of Type II Hybrid Arch Repair for Complex Aortic Arch Pathology

**DOI:** 10.3389/fcvm.2022.882783

**Published:** 2022-06-02

**Authors:** Yanxiang Liu, Bowen Zhang, Shenghua Liang, Yaojun Dun, Hongwei Guo, Xiangyang Qian, Cuntao Yu, Xiaogang Sun

**Affiliations:** Department of Vascular Surgery, State Key Laboratory of Cardiovascular Disease, Fuwai Hospital, National Center for Cardiovascular Diseases, Chinese Academy of Medical Sciences and Peking Union Medical College, Beijing, China

**Keywords:** hybrid arch repair (HAR), aortic arch pathology, aortic dissection (AD), arch aneurysm, deep hypothermic circulatory arrest

## Abstract

**Background:**

The hybrid arch repair (HAR) is an appealing surgical option in the management of aortic arch diseases. The aim is to evaluate the short and mid-term outcomes of type II HAR involving replacement of the ascending aorta, arch debranching, and zone 0 stent graft deployment in diverse arch pathologies.

**Methods:**

200 patients with various diffuse aortic pathologies involving the arch were enrolled between 2016 and 2019. Complex arch diseases included acute type A dissection (*n* = 129, 64.5%), acute type B dissection (*n* = 16, 8.0%), aortic arch aneurysm (*n* = 42, 21.0%) and penetrating arch ulcer (*n* = 13, 6.5%). Mortality, morbidity, survival and re-intervention were analyzed.

**Results:**

The overall 30-day mortality rate was 8.0% (16/200). Stroke was present in 3.5% (7/200) of the general cohort and spinal cord injury was occurred in 3.0% (6/200). Multivariable logistic analysis showed that cardiac malperfusion and CPB time were the risk factors associated with 30-day mortality. The mean follow-up duration was 25.9 months (range 1–57.2 months), and the 3-year survival rate was 83.1%. On Cox regression analysis, age, diabetes, cardiac malperfusion and CPB time predicted short and mid-term overall mortality. A total of 3 patients required reintervention during the follow-up due to the thrombosis of epiaortic artificial vessels (*n* = 1), anastomotic leak at the site of the proximal ascending aorta (*n* = 1) and the type I endoleak (*n* = 1).

**Conclusions:**

Type II HAR was performed with satisfactory early and mid-term outcomes in complex aortic arch pathologies.

## Introduction

Management of aortic arch aneurysm and dissection remains challenging. Open total arch replacement for complex arch diseases requires the use of hypothermic circulatory arrest and adjunct cerebral protection strategies. Due to the complexity of this operation, patients with advanced age or multiple comorbidities may experience significant morbidity and mortality (1). With the development of endovascular technology, hybrid arch repair (HAR) has become an alternative surgical option in patients with complex aortic arch pathologies, especially in the high risk population (2). Combining conventional surgical techniques with endovascular technology, HAR limits the duration of hypothermic circulatory arrest and cerebral ischemia by simplifying and shortening the arch repair procedure, thus minimizing the operation (3–5).

Based on the aortic arch anatomy, the required hybrid arch operative technique may vary. Therefore, HAR is classified into three major types, I, II and III (6). For the complex arch pathologies complicated with the ascending and descending thoracic aorta lesions, the type II HAR is the optimal choice, which involves replacement of the ascending aorta, arch debranching, and zone 0 stent graft deployment (7–9).

This series presents the early and midterm outcomes of our type II hybrid arch procedure. Our goal was to assess the outcome of this operation when performed in diverse arch pathologies.

## Patients and Methods

### Patients

Between January 2016 and December 2019, 780 patients with complex aortic arch diseases underwent total arch replacement (frozen elephant trunk or type II HAR). Of those, a total of 212 patients underwent type II HAR in our institute. 12 patients with subacute/chronic aortic dissection (>14 days of onset) were excluded from this cohort due to their unique clinical manifestations (10). The remaining 200 patients were divided into acute aortic dissection (AAD) group including acute type A dissection (*n* = 129, 64.5%) and acute type B dissection (*n* = 16, 8.0%), and thoracic aortic aneurysm (TAA) group including aortic arch aneurysm (*n* = 42, 21.0%) and penetrating arch ulcer (*n* = 13, 6.5%). This retrospective study was approved by the ethics committee of Fuwai Hospital, and the consent of patients was waived.

### Indications and Imaging

**Figure 1:**
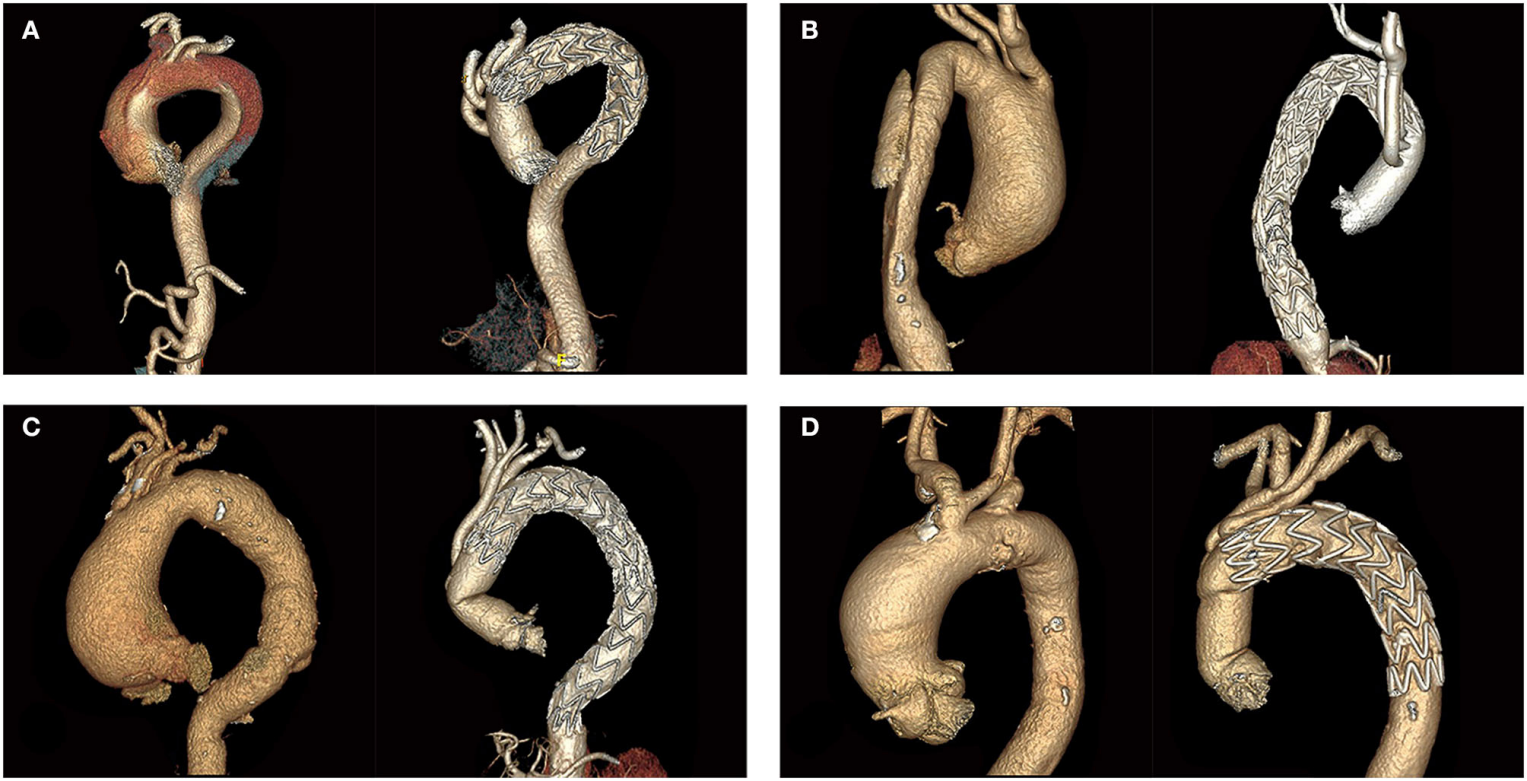
Preoperative and postoperative CT scans of different aortic arch diseases. **(A)** Acute type A aortic dissection. **(B)** Acute type B aortic dissection. **(C)** Aortic arch aneurysm. **(D)** Penetrating arch ulcer.

These 200 patients were thought to be at prohibitively high risk of conventional repair, and then underwent HAR. All standards used for surgical planning were relative factors, and were not absolute indications for HAR. The selection of the type of aortic repair was made at the discretion of surgeons. Advanced age (age > 60 years) and significant comorbidities such as previous cerebrovascular disease, pulmonary dysfunction, or left ventricular dysfunction were relevant factors favoring HAR. Malperfusion syndrome was not a factor to be considered. In our experience, most malperfusion syndromes present as dynamic ischemia. Both open arch repair (especially total arch replacement with frozen elephant trunk) and HAR can expand the true lumen, and then restore the perfusion. For acute type A dissection, type II HAR was performed regardless of the location of the primary intimal tear. If complicated with ascending aorta lesions, patients with acute type B dissection and arch aneurysm (ulcer) were also treated with type II HAR. This is because zone 0 stent graft proximal landing in diseased ascending aorta increased the risk of endoleak and retrograde type A dissection. The standard for ascending aorta replacement in our center is the diameter >40 mm. Preoperative and postoperative CT scan images for the different pathologies treated are shown in Figure 1.

### Operative Technique

The surgical procedure has been described previously in detail (9, 11, 12). Cerebrospinal fluid drainage is not routinely performed preoperatively. Patients with spinal cord injury in the early postoperative period had cerebrospinal fluid drainage at the bedside in the intensive care unit and underwent appropriate anticoagulation, measures to improve mean arterial pressure, and other treatments.

The operation was performed in the hybrid operating room equipped with a fixed C-arm fluoroscopy system. All these patients were placed on cardiopulmonary bypass (CPB). Nasopharyngeal temperature was lowered to 28°C in all cases. The arterial cannulation was performed via the right axillary and the femoral arteries. The femoral artery was cannulated for the perfusion of the lower body during the arch anastomosis. With an aortic cross clamp placed on the distal ascending aorta, aortic root or valve procedures were performed if necessary. Then a 4-branched prosthesis graft was anastomosed to the sinotubular junction to replace the ascending aorta. Subsequently, the aortic cross-clamp was used between the innominate artery and the left common carotid artery. The aortic arch was transected proximal to the left common carotid artery. The distal end of the graft was then sutured end-to-end to the aortic arch. The arch vessel debranching was performed individually, starting with the left common carotid artery. The endograft was delivered in a retrograde fashion through the incision of the original femoral cannulation. Its proximal end was anchored to the prosthetic graft to complete the arch repair. The stent graft was oversized by 10 to 20%.

### Follow-Up

Of 184 patients who survived beyond the early postoperative period, 182 (98.9%) were successfully followed up during outpatient visits or by telephone. Moreover, postoperative follow-up CT scans were performed for all patients upon discharge, and further imaging assessments were scheduled at 3 and 6 months postoperatively and annually thereafter.

### Statistical Analysis

Continuous data are presented as the mean ± standard deviation or median with an interquartile range (IQR) and were analyzed with Independent *t*-test or the Mann-Whitney U-test, as appropriate. Categoric variables are reported as counts and percentages and were compared using the Pearson χ^2^ test or Fisher's exact test.

A multivariable logistic regression analysis was performed to evaluate risk factors for 30-day mortality and stroke. Covariates in the regression models included AAD, 16 preoperative variables in Table 1, redo sternotomy, emergency surgery, Bentall procedure, coronary artery bypass grafting, CPB time and cross-clamp time. A forward stepwise variable selection approach was performed.

**Table 1:** Preoperative data.

**Variables**	**Overall (*n* = 200)**	**AAD (*n* = 145)**	**TAA (*n* = 55)**	***P*-value**
Age (years)	61.8 ± 7.5	61.5 ± 7.5	62.8 ± 7.3	0.275
Male	132 (66.0)	93 (64.1)	39 (70.9)	0.367
Body mass index	25.8 ± 3.8	25.9 ± 3.9	25.6 ± 3.6	0.682
Hypertension	169 (84.5)	130 (89.7)	39 (70.9)	0.001
Coronary artery disease	47 (23.5)	29 (20.0)	18 (32.7)	0.058
Diabetes	16 (8.0)	9 (6.2)	7 (12.7)	0.220
COPD	8 (4.0)	5 (3.4)	3 (5.5)	0.808
Cerebrovascular event	17 (8.5)	15 (10.3)	2 (3.6)	0.217
Chronic kidney disease	2 (1.0)	2 (1.4)	0 (0.0)	1.000
NYHA≥3	8 (4.0)	7 (4.8)	1 (1.8)	0.572
**Organ malperfusion**				
Cardiac	6 (3.0)	6 (4.1)	0 (0.0)	0.286
Cerebral	7 (3.5)	7 (4.8)	0 (0.0)	0.219
Visceral	4 (2.0)	4 (2.8)	0 (0.0)	0.497
Limb	1 (0.5)	1 (0.7)	0 (0.0)	1.000
LVEF	60.4 ± 4.8	60.1 ± 5.1	60.1 ± 4.0	0.198
Median or massive AR	31 (15.5)	22 (15.2)	9 (16.4)	0.835

*COPD, chronic obstructive pulmonary disease; NYHA, New York Heart Association; LVEF, left ventricular ejection fraction*.

Death at follow-up was analyzed with the Kaplan-Meier method and Cox proportional-hazard risk model. The covariates and variable selection approach for the Cox model was the same as the logistic regression analysis.

A 2-tailed *P*-value < 0.05 indicated statistical significance. R version 3.6.3 (The R Foundation for Statistical Computing) was used for analysis of survival. Other statistics were analyzed using SPSS version 25 (IBM, Armonk, NY).

## Results

### Baseline Characteristics and Operative Data

Patients' preoperative characteristics are shown in Table 1. Patients' mean age was 61.8 ± 7.5 years, and 132 patients were male (66.0%). In patients with AAD, the rate of hypertension was higher (89.7 vs. 70.9%, *P* = 0.001). A higher proportion of coronary artery disease was observed in the patients with TAA, but there was no significant difference between the two groups (32.7 vs. 20.0%, *P* = 0.058). Preoperative malperfusion syndrome occurred in 12.4% of patients in the AAD group (18/145).

More patients in the AAD group underwent emergency surgery (57.2 vs. 9.1%, *P* < 0.001). Sinus reconstruction was performed in 40.0% of patients in the AAD group (58/145). The cross-clamp time were significantly longer in the AAD group (*P* = 0.038).

There was no significant difference in CPB time between the two groups. The operative data are presented in Table 2.

**Table 2:** Operative details.

**Variables**	**Overall**	**AAD**	**TAA**	***P*-value**
	**(*n* = 200)**	**(*n* = 145)**	**(*n* = 55)**	
Redo sternotomy	7 (3.5)	5 (3.4)	2 (3.6)	1.000
Emergency surgery	88 (44.0)	83 (57.2)	5 (9.1)	<0.001
**Combined surgery**				
Sinus reconstruction	58 (29.0)	58 (40.0)	0 (0.0)	<0.001
Bentall	24 (12.0)	16 (11.0)	8 (14.5)	0.495
CABG	41 (20.5)	25 (17.2)	16 (29.1)	0.064
AVR	13 (6.5)	6 (4.1)	7 (12.7)	0.060
Other (David, Mitral, Congenital)	4 (2.0)	2 (1.4)	2 (3.6)	0.651
CPB time (min)	137.0 (59.0)	137.0 (63.0)	137.0 (57.0)	0.256
Cross-clamp time (min)	78.0 (52.0)	82.0 (53.0)	66.0 (37.0)	0.038

*Emergency surgery is defined as surgery performed within 24 h after the patient's emergency visit in our center*.

### Early Outcomes

The overall 30-day mortality rate was 8.0% (16/200). Causes of death were multiorgan failure in 3.5% (*n* = 7), low cardiac output in 1.0% (*n* = 2), septic shock in 1.0% (*n* = 2), respiratory insufficiency in 1.0% (*n* = 2), fatal neurologic event in 0.5% (*n* = 1), aortic rupture in 0.5% (*n* = 1) and cardiac arrest in 0.5% (*n* = 1).

Stroke, defined as any new global or focal neurologic deficit that was clinically or radiographically evident, was present in 3.5% (7/200) of the general cohort. In the overall population, spinal cord injury corresponded with any new lower extremity deficit unrelated to an intracerebral event and was occurred in 3.0% (6/200).

The in-hospital time in the TAA group was significantly longer than that in the AAD group (*P* < 0.001), which can be explained by the long hospital stay for preoperative examination in the patients with TAA. Patients with AAD had longer ventilation time (*P* = 0.007). The overall prevalence of postoperative complications is shown in Table 3.

**Table 3:** Early postoperative outcomes.

**Variables**	**Overall (*n* = 200)**	**AAD (*n* = 145)**	**TAA (*n* = 55)**	***P*-value**
30-day mortality	16 (8.0)	13 (9.0)	3 (5.5)	0.599
Stroke	7 (3.5)	6 (4.1)	1 (1.8)	0.714
Spinal cord injury	6 (3.0)	5 (3.4)	1 (1.8)	0.889
Dialysis	18 (9.0)	15 (10.3)	3 (5.5)	0.422
Hepatic dysfunction	9 (4.5)	7 (4.8)	2 (3.6)	1.000
Reintubation or tracheotomy	10 (5.0)	6 (4.1)	4 (7.3)	0.586
Low cardiac output syndrome	7 (3.5)	5 (3.4)	2 (3.6)	1.000
ICU time (h)	92.9 (94.9)	93.3 (113.2)	83.9 (90.9)	0.150
In-hospital time (d)	15.0 (8.0)	14.0 (5.0)	21.0 (14.0)	<0.001
Ventilation time (h)	21.5 (24.2)	22.9 (26.4)	16.4 (20.9)	0.007
Ventilation time >48 h	43 (21.5)	35 (24.1)	8 (14.5)	0.140

### Multivariable Logistic Regression Analysis

Multivariable analysis showed that cardiac malperfusion and CPB time were factors associated with 30-day mortality. And CPB time was also identified risk factor for stroke. The results of the logistic regression analysis are shown in Table 4.

**Table 4:** Multivariable logistic regression analysis results for 30-day mortality and stroke.

**Factors**	**OR**	**95% CI**	***P*-value**
**30-day mortality**			
Cardiac malperfusion	18.748	3.214–109.348	0.001
CPB time	1.009	1.004–1.015	0.001
**Stroke**			
CPB time	1.011	1.004–1.018	0.003

*OR, odds ratio; CI, confidence interval*.

### Mid-Term Survival and Reintervention

**Figure 2:**
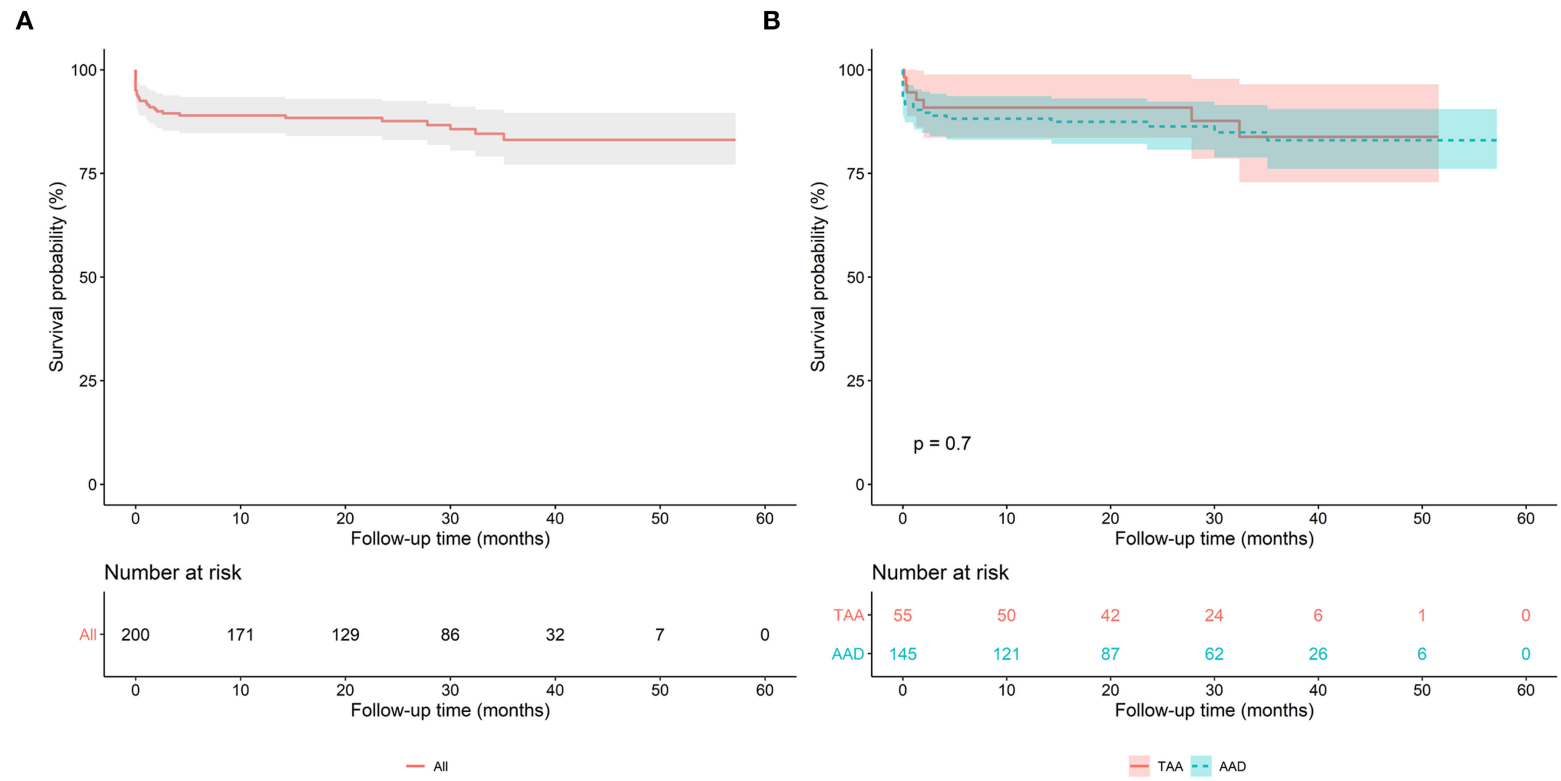
**(A)** Kaplan-Meier analysis of the overall population after the FET technique. **(B)** Kaplan-Meier analysis of the acute aortic dissection (AAD) group and the thoracic aortic aneurysm (TAA) group.

The mean follow-up duration was 25.9 months (range 1.0–57.2 months). Postoperative death during follow-up was seen in 12 patients. The causes of death were as follows: five pneumonia and respiratory failure, two multiple organ failure, two cerebrovascular accident, one aortic event, one renal failure, and one acute myocardial infarction. The survival at 1 and 3 years was 89.0 and 83.1%, respectively, as shown in Figure 2A. No significant difference was found in the survival rate of patients with AAD and patients with TAA (*P* = 0.7), as presented in Figure 2B. On Cox regression analysis, age, diabetes, cardiac malperfusion and CPB time predicted short and mid-term overall mortality (Table 5).

**Table 5:** Multivariable Cox-hazard regression analysis results for short and mid-term overall mortality.

**Factors**	**HR**	**95% CI**	***P*-value**
Age	1.063	1.009–1.119	0.021
Diabetes	3.799	1.502–9.608	0.005
Cardiac malperfusion	6.618	2.136–20.509	0.001
CPB	1.008	1.004–1.011	<0.001

*HR, hazard ratio; CI, confidence interval*.

A total of 3 patients required reintervention due to the thrombosis of epiaortic artificial vessels (*n* = 1), anastomotic leak at the site of the proximal ascending aorta (*n* = 1) and the type I endoleak (*n* = 1).

### Radiological Follow Up

A follow-up CT scan was available in 181 of 184 survivors. Patients with a follow-up period of <12 months were excluded from imaging analysis. 85 patients, including 60 patients with AAD and 25 patients with TAA, were followed up for more than 12 months with an average of 19.3 months (range 12.0–48.0 months).

Complete false lumen thrombosis of peri-stent aorta was achieved in 100% (60/60) in the AAD patients. Endoleak, stent migration, stent deformation, or SINE (Stent-induced new entry) was not found in any patients beyond 12 month follow-up. Of note, the artificial blood vessel occlusion of the left subclavian artery or left common carotid artery appeared in five patients.

## Comment

Complex diffuse aortic pathology involving the arch remains a clinical challenge. With the advanced stent-graft technology available, the single-stage type II HAR becomes an attractive alternative to conventional open repair in various diseases of the aortic arch. However, few data are currently available concerning the type II HAR, either because of the small size of the studies (13, 14), or because of the mixed cases of different hybrid procedures (I and II) and landing zones (zone 0 - zone 2) in the studies (15–17). To the authors' knowledge, with a total of 200 patients included, this study is the largest series of type II HAR with zone 0 stent graft deployment. Its goal is to clarify the value of the type II HAR for diverse aortic arch pathologies in terms of short and mid-term results.

Stent-related complications are a concern. Type I endoleak, rupture, pseudoaneurysm formation, and retrograde type A dissection can be the consequence of stent deployment in a diseased, native aorta. Type I endoleak is reported in 15 to 30% of cases (18–21). And the devastating complication of acute retrograde type A dissection may be as high as 6% (18). Therefore, type I hybrid surgeries with zone 0 stent graft deployment were rarely performed in our center. In the past 10 years, only 36 patients underwent type I HAR with the stent graft anchored to the native ascending aorta. Joseph E. Bavaria performed type II HAR in patients with ascending aorta diameter >37 mm, which was more aggressive than our standard (22). In our study, only three patients needed further intervention. The reoperation-free survival at 5 years in the patients with aortic dissection was about 93.0% in our previous type II HAR studies (12, 23). This good mid-term result was mainly due to the replacement of the ascending aorta providing a safer landing zone for stent graft deployment, and then reducing complications resulted from the proximal deployment in a native diseased aorta.

In general, for patients undergoing a type II repair, a short period of deep hypothermic circulatory arrest (DHCA) was used to perform an open distal anastomosis (6, 13). In our center, the aortic arch was clamped between the innominate artery and the left common carotid artery when doing the arch anastomosis, and perfusion of the lower body was through the femoral artery to avoid DHCA.

Avoiding DHCA is the biggest advantage of this more minimally invasive surgery compared to the frozen elephant trunk (FET), which is another attractive approach to treat the multisegmental aortic disease in the present day. The FET technique also allows single-stage repair of extensive aortic disease. What' more, it combines the durability of an open arch replacement with the benefit of a stent graft insertion into the descending aorta.

Theoretically, with relatively easy surgical operation and the avoidance of DHCA, type II HAR is expected to show superiority compared with the FET procedure. However, two studies worthy of attention did not found the differences in the mortality, morbidity, and the survival and re-intervention rates between the two procedures. Liang Zhang and his colleagues compared the type II HAR and FET procedure in DeBakey type I aortic dissection, and found no significant difference in the early death, postoperative complications, and mid-term survival and freedom from reoperation between the two groups (23). A comparative study of zone 0 hybrid arch exclusion versus traditional open repair performed by Ourania Preventza found that adverse outcomes were not significantly different between the two surgeries and were more related to the preoperative comorbidities rather than the procedure type (hybrid or traditional) (24).

The authors believe that at present, conventional open treatment of aortic arch disease with total arch replacement still remains the gold standard, which may not only be in low-risk patients. A recent study showed that open total arch replacement was performed with an acceptable overall survival in octogenarians with 30-day mortality 8.6% comparable to the 8.0% in our study (25). We think that HAR shows its real merits in some patients with special anatomical characteristics and then serve as a complement for FET. For example, in the setting of AAD, the true lumen of the descending aorta is sometimes severely and extensively compressed. Then the surgeon can deploy additional stent grafts or bare metal stents during HAR, if the fluoroscopy reveals the malperfusion of distal aorta, visceral or renal vessels. In addition, extent of the descending aortic coverage can be individualized in HAR, which facilitates the sealing of distal entries in the descending aorta. For TAA, if the distal arch aneurysm is large and there is not enough landing zone for FET, the stent graft in HAR can be tailored and anchored to the descending aorta to prevent type Ib endoleak. From our perspectives, the greatest significance of hybrid surgery is to provide a brand-new alternative for aortic diseases, and to bridge the gap between conventional open surgery and total endovascular repair.

### Limitations

This was a single center retrospective study with relatively short follow-up time. These procedures were performed by the experienced aortic surgeons, and the results may not be translatable to all hospitals.

## Conclusions

Type II HAR was performed with satisfactory early and mid-term results in acute and chronic diffuse thoracic aortic pathologies involving the arch. Over the years, the stent graft has been approaching the heart step by step, starting from the descending aorta, passing through the aortic arch, and entering into the ascending aorta. Before the connection between endovascular technology and TAVI (Transcatheter Aortic Valve Implantation) technology, long-term follow-up is required to help us fully understand the true impact of stent deployment into the ascending aorta.

## Data Availability Statement

The raw data supporting the conclusions of this article will be made available by the authors, without undue reservation.

## Ethics Statement

This retrospective study was approved by the ethics committee of Fuwai Hospital, and the requirement for the written informed consent of patients was waived.

## Author Contributions

YL wrote the first draft of the manuscript. YL and BZ performed the statistical analysis. SL organized the database. YD and HG contributed to conception and design of the study. XQ, CY, and XS provided the data. All authors contributed to manuscript revision, read, and approved the submitted version.

## Funding

This work was supported by the Beijing Municipal Science and Technology Commission (Z181100001718197).

## Conflict of Interest

The authors declare that the research was conducted in the absence of any commercial or financial relationships that could be construed as a potential conflict of interest.

## Publisher's Note

All claims expressed in this article are solely those of the authors and do not necessarily represent those of their affiliated organizations, or those of the publisher, the editors and the reviewers. Any product that may be evaluated in this article, or claim that may be made by its manufacturer, is not guaranteed or endorsed by the publisher.

## Abbreviations

HAR: hybrid arch repair
CPB: cardiopulmonary bypass
AAD: acute aortic dissection
TAA: thoracic aortic aneurysm
CABG: coronary artery bypass graft
CT: computed tomography
IQR: interquartile range
DHCA: deep hypothermic circulatory arrest
FET: frozen elephant trunk.
